# Beyond the cost‐effectiveness acceptability curve: The appropriateness of rank probabilities for presenting the results of economic evaluation in multiple technology appraisal

**DOI:** 10.1002/hec.3884

**Published:** 2019-05-02

**Authors:** David Epstein

**Affiliations:** ^1^ Department of Applied Economics University of Granada Granada Spain

**Keywords:** cost‐effectiveness acceptability curve, decision‐making, decision model, health technology appraisal, rank probabilities

## Abstract

The cost‐effectiveness acceptability curve (CEAC) shows the probability that an option ranks first for net benefit. Where more than two options are under consideration, the CEAC offers only a partial picture of the decision uncertainty. This paper discusses the appropriateness of showing the full set of rank probabilities for reporting the results of economic evaluation in multiple technology appraisal (MTA). A case study is used to illustrate the calculation of rank probabilities and associated metrics, based on Monte Carlo simulations from a decision model. Rank probabilities are often used to show uncertainty in the results of network meta‐analysis, but until now have not been used for economic evaluation. They may be useful decision‐making tools to complement the CEAC in specific MTA contexts.

## 1 INTRODUCTION

Standard decision rules for economic evaluation calculate incremental cost‐effectiveness ratios (ICER), excluding dominated and extendedly dominated options. Uncertainty is shown using the cost‐effectiveness acceptability curve (CEAC). These decision rules assume that only one option (that with the greatest mean net benefit) will be chosen by the decision maker, and all the others will be rejected. This assumption may be inappropriate for some health technology appraisal (HTA) settings where multiple options are compared. Although an individual patient can only be given one option, there may be specific circumstances where the decision maker does not wish to recommend only one option for the entire patient population, for example, where the policy maker wants to encourage competition, or aims to diversify risk.

A risk neutral decision maker who had to choose between two options would prefer that with greater mean net benefit, and would not be concerned by the chance that this option may give lower net benefit (Claxton, 1999). In practice, decision makers are sometimes concerned about these risks, for example because they are not risk neutral, or because they may want to make a provisional decision conditional on further evidence (Rothery et al., 2017). Where there are multiple options, it may be of interest to estimate the probability that a treatment ranks second, or third, and so on. This article deals with the situation where a choice needs to be made between a finite number of alternative therapies for a specific patient group. However, the ranks must not be interpreted as identifying an optimal “sequence” of therapies (first‐line, second‐line, etc.).

It is noteworthy that reporting guidelines for network meta‐analysis (NMA) state that “authors are encouraged to report not only the probability of each intervention being best, but also a more complete presentation of rankings that includes the probability of being at least second best, third best, etc. This provides a picture of the uncertainty associated with the rankings” (Hutton et al., 2015). To better communicate uncertainty, authors for NMA also recommend graphical tools such as rankograms (Chaimani, Higgins, Mavridis, Spyridonos, & Salanti, 2013; Hutton et al., 2015)_._ So far, such measures have not been used for presenting the results of economic evaluation.

Therefore, there may be circumstances where rank probabilities are useful as decision‐making tools alongside traditional metrics such as ICER and CEAC. This article shows how rank probabilities for net benefit are calculated, how they are related to the CEAC, and discusses how decision makers might act on these. Furthermore, this article argues that displaying the full set of rank probabilities, alongside the cost‐effectiveness plane and the CEAC, can assist communication between health economists and decision makers and help explain some well‐known paradoxes that sometimes arise in the economic evaluation of multiple technology appraisal (MTA), such as when a dominated option can be most likely to have the greatest net benefit.

## 2 METHODS

### 2.1 Rank probabilities

Suppose there are *J* mutually exclusive treatment options, and, using a probabilistic decision model, the mean costs *C*_*j*_ and quality‐adjusted life years (QALYs) *Q*_*j*_ of each option *j* are estimated by Monte Carlo simulation, along with mean net benefit, *NB*_*j*_ = *λQ*_*j*_ *– C*_*j*_. The rank probability *P*_*jr*_ is the probability that option *j* takes rank *r* in terms of net benefit, for *j* = 1..*J* and *r* = 1..*J*. Thus, the CEAC shows the probability *P*_*j1*_. The cumulative rank probability $P_{\mathrm{jr}}^{\mathrm{cum}}\;$is the probability that option *j* is up to a particular rank *r*.

The rank probabilities (*P*_*j2*_ *, P*_*j3*_ *,.., P*_*jr*_ *,* up to *P*_*jJ*_) can be easily derived (along with the CEAC) from the results of Monte Carlo simulations, using the rank() function in Excel for example. The rank probabilities can be displayed graphically as rankograms or cumulative rankograms (Salanti, Ades, & Ioannidis, 2011).

Guidelines for NMA also recommend summary measures such as the surface under the cumulative ranking, or SUCRA, defined as $\sum_{r=1}^{J-1}P_{\mathrm{jr}}^{\mathrm{cum}}$/*(J‐1)* (Chaimani et al., 2013; Hutton et al., 2015)_._ This is interpreted in the NMA literature as the percentage of rank that each option has, compared with an ideal treatment that always ranks first without uncertainty (Chaimani et al., 2013). However, as the SUCRA is an unweighted average of the cumulative rank probabilities, it lacks any empirical or theoretical grounding (such as utility theory) for preference‐based choice. Therefore, it does not seem an appropriate summary measure for use in economic evaluation, and this article focuses on the use of rank probabilities as measures of uncertainty alongside the CEAC.

### 2.2 Case study

A case study is used to illustrate and motivate the discussion. The patients are people with severe symptomatic varicose veins (National Clinical Guideline Centre, 2012). The costs and QALYs were generated by a stochastic model, available at https://doi.org/10.17632/pmrt2gnzcr.1. There are seven therapies, labeled A–G. A is the standard interventional treatment, taken as the reference or “comparator.” B is conservative care, that is, no interventional treatment, with no initial cost. The others are competing interventional therapies. The input data to the model are stylized but are intended to be fairly realistic (Epstein, Onida, Bootun, Ortega‐Ortega, & Davies, 2018), see Table S1.

## 3 RESULTS

### 3.1 ICER and CEAC

**Figure 1:**
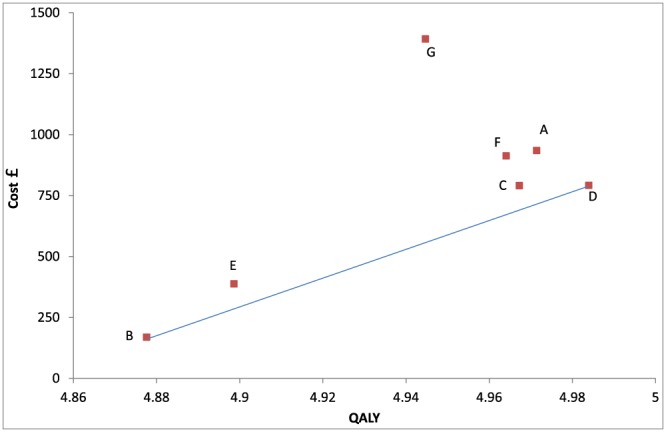
Results of the varicose veins case study: total mean cost and mean quality‐adjusted life year per person for each strategy, and the efficiency frontier [Colour figure can be viewed at http://wileyonlinelibrary.com]

**Figure 2:**
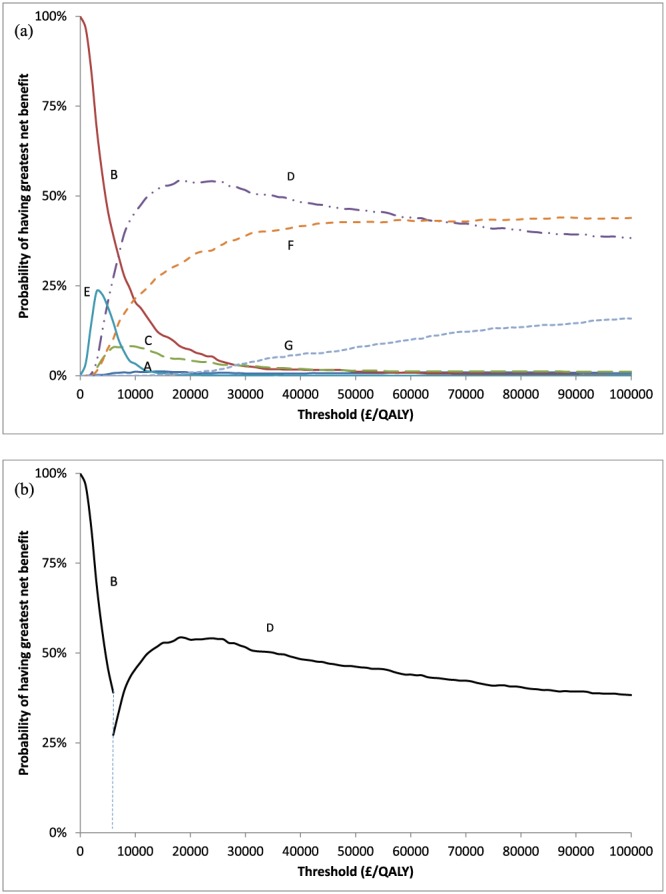
Results of the varicose veins case study: (a) cost‐effectiveness acceptability curves and (b) cost‐effectiveness acceptability frontier [Colour figure can be viewed at http://wileyonlinelibrary.com]

The ICER of B versus D was £ 5,857/QALY. All other options were dominated or extendedly dominated (Figure 1). Hence, D has the greatest mean net benefit for all λ > £ 5,857/QALY. The cost‐effectiveness acceptability frontier shows, at given λ, the probability that the option with the highest mean net benefit is the highest ranked for net benefit (Fenwick, Claxton, & Sculpher, 2001). At λ = £20,000, D had the greatest mean net benefit and was the option with the highest probability (54%) to have the greatest net benefit (Figure 2 and Table 1). Option C has a very low CEAC probability at λ = £20,000 (4%), but has the second highest mean net benefit (Table 1). Option F was dominated, and thus will never have the greatest mean net benefit at any λ (Barton, Briggs, & Fenwick, 2008) but nevertheless had a relatively high probability (33%) of having greatest net benefit. At λ above £65,000, F was most likely to have the greatest net benefit (Figure 2).

**Table 1:** Rank of net benefit for each treatment for the varicose veins case study at a threshold λ of £20,000/QALY

	D	F	C	A	G	B	E
Rank probability							
(CEAC) 1	54%	33%	4%	1%	1%	7%	0%
2	33%	25%	21%	11%	7%	4%	0%
3	9%	6%	38%	31%	11%	3%	1%
4	3%	9%	23%	39%	15%	5%	7%
5	1%	9%	12%	16%	20%	13%	28%
6	0%	10%	3%	3%	16%	27%	42%
7	0%	8%	0%	0%	29%	41%	22%
Cumulative rank probability							
1	54%	33%	4%	1%	1%	7%	0%
2	86%	58%	25%	12%	8%	11%	1%
3	96%	63%	63%	43%	19%	15%	2%
4	99%	72%	86%	81%	35%	19%	9%
5	100%	81%	98%	97%	55%	33%	36%
6	100%	92%	100%	100%	71%	60%	78%
7	100%	100%	100%	100%	100%	100%	100%
SUCRA	89%	66%	63%	56%	31%	24%	21%
Rank of mean net benefit	1	4	2	3	5	7	6
Mean rank	1.7	3.0	3.3	3.7	5.1	5.6	5.7
Median rank (IQR)	1 (1–2)	2 (1–5)	3 (3–4)	4 (3–4)	5 (4–7)	6 (5–7)	6 (5–6)

*Note*. The options have been ordered in the table from highest to lowest mean rank, calculated as ∑^*J*^_*r = 1*_ *(P*_*jr*_ *× r)*. The first row of percentages corresponds to the CEAC probabilities for a threshold λ = £20000/QALY.

Abbreviations: CEAC, cost‐effectiveness acceptability curve; SUCRA, surface under the cumulative ranking; QALY, quality‐adjusted life year.

### 3.2 Rank probabilities

It is well known that an option with the greatest mean net benefit might not be that with greatest probability of highest net benefit, and dominated or extendedly dominated options can have the greatest probability of highest net benefit (Barton et al., 2008). Hence, superficially, the ICERs and the CEACs can present a somewhat contradictory message, even though they are calculated from the same data. Barton et al. showed that this situation arises when multiple options are correlated and there are differences in the level of variation of net benefit between them (Barton et al., 2008).

However, the CEAC reports a partial picture of the uncertainty. It shows the probability that option *j* has the greatest net benefit, but not the variance in net benefit. Showing policy makers the full table of rank probabilities can help explain these apparent paradoxes. Option F had a relatively high probability of being the most cost‐effective (as shown by the CEAC), but also a relatively high probability of a low rank (which is not apparent from the CEAC). This variance was also seen in the inter‐quartile range (IQR) of the ranks. Option F ranked first in >25% of simulations and fifth or worse in >25% of simulations. Other therapies had a tighter IQR, indicating more certainty about their relative position. Showing decision makers the rank probabilities allows them to observe this variation and hence, hopefully, better understand the decision model.

### 3.3 Rank of treatments

If the decision maker wishes to rank treatments and is concerned both about mean net benefit and about the chance a treatment has lower net benefit, then there may be no unambiguously “correct” order. The SUCRA is a summary measure that compares whether one option is more likely to rank higher overall than another. The SUCRA for F is 66% and that for C is 63% at λ = £20,000/QALY (Table 1). But, this does not mean F will be or should be preferred to C. F is more likely than C to rank first or second, but equally likely to rank in the top three, and less likely to rank in the top four. Whether F is preferred to C depends on the importance or weight the decision maker gives to each place in the rank. Hence, unweighted aggregate measures of rank such as the SUCRA, mean rank, or median rank are unsuitable for preference‐based choices.

**Figure 3:**
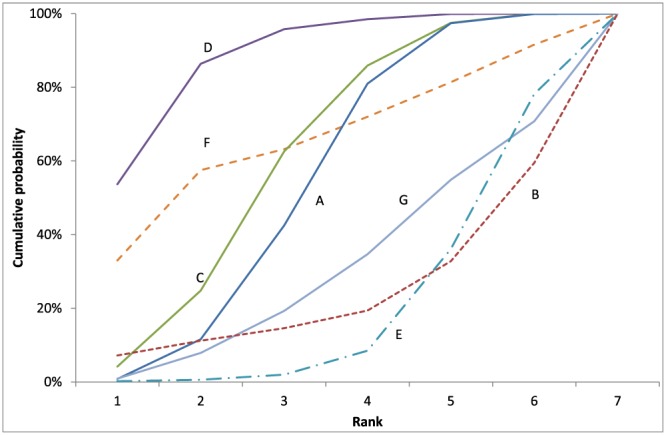
Varicose veins case study: cumulative rank probabilities (cumulative rankograms) for each option [Colour figure can be viewed at http://wileyonlinelibrary.com]

First‐order stochastic dominance (FSD) can be a useful tool to place some options in order of preference, without requiring any information about the decision maker's attitude to risk (Leshno & Levy, 2004). One option shows FSD over another when it has a higher or equal probability of being up to any rank *r*, at a given threshold λ. So, for example, Option D shows FSD over all the others (Figure 3). Hence, D should be unambiguously preferred to any other option. In the same way, FSD can unambiguously place options D≽C≽A≽G in that order. Likewise, FSD indicates D≽F and F≽G, but the absolute position of F could be second, third, or fourth, depending on the decision maker's attitude to risk. In the same way, a decision maker might place B, G, and E in any order. Examination of the interquartile ranges of rank in Table 1 shows greater variability in the ranks of F, G, and B than can be seen in other options.

## 4 DISCUSSION

The appropriate results to report in an economic evaluation will depend on the decision maker's objectives. Even formalized HTA agencies like National Institute for Health and Care Excellence (NICE) and Institute for Quality and Efficiency in Health Care (IQWiG) in practice use different and often evolving paradigms depending on context. Given a particular decision problem, what information might usefully support the decision maker?

In some MTA, the decision maker may wish to approve the option with the greatest mean net benefit and reject the others. In this paradigm, the cost‐effectiveness plane and the CEAC would offer appropriate information, with the CEAC indicating the degree of uncertainty about which option offered greatest net benefit (the other ranks being irrelevant to the decision maker). Rank probabilities would not be useful for this decision maker.

However, this “first past the post” policy is not the only possible decision‐making paradigm. It assumes the decision maker gives no importance at all to the chance the option may be second best, third best, and so forth. In the case study, the decision maker may welcome the knowledge that, at the given threshold λ, Option D not only offers greatest mean net benefit, and has the highest probability of greatest net benefit (CEAC), but also has the highest probability of being up to any rank (FSD). There may also be circumstances where the “first‐best” option identified by the analyst is ruled out for some reason (for example, it is subsequently withdrawn from the market by the manufacturer). In such cases, information about the second‐best option would be useful to make a decision.

Reporting the complete matrix of rank probabilities may help the decision maker understand the decision model better, by indicating the degree of variance in the rankings. In the case study, Option F (a dominated therapy) had a high probability of greatest rank, but also a high probability of low rank. This variance in net benefit is not revealed from the CEAC alone. From this information, Option F might be characterized as a “promising” innovative treatment, one with a good chance of success but also a high chance of failure.

Rank probabilities can be useful in economic evaluation in situations where the decision maker wishes to recommend more than one option. The fact that options are “mutually exclusive” at an individual level does not preclude an HTA body approving reimbursement for multiple options at a population level. Manski refers to this as the ability to “socially diversify” risks that are individually indivisible (Manski, 2018). There may be several examples. First, if the decision maker wishes to fund a promising but uncertain treatment (such as F) to promote “learning‐by‐doing,” as well as funding mature and cost‐effective treatments (such as D). Second, if the central HTA agency is responsible for approving therapies onto a “positive list” for reimbursement, leaving the final choice to clinicians and patients. Third, the HTA agency may wish to encourage competition in the health‐care market (Cole & Dusetzina, 2018; Danzon & Chao, 2000; Guha, Lacy, & Woodhouse, 2008; Morton & Boller, 2017; United Nations, 2015). Even in tightly regulated European pharmaceutical markets, the notified reimbursement price is often a “maximum,” and manufacturers negotiate discounts with local procurement agencies. The negotiating power of local buyers may be augmented if a range of substitutes are reimbursed. Likewise, some medical devices markets are characterized by acute competition and rapid technological advance, and the role of a central HTA agency may be to provide information (rather than prescriptive guidance) to local decision makers.

If the decision maker wishes to recommend *r* options, then it might be useful to know the probability that an option is in the top *r*. For example, in the case study, the HTA agency might wish to provide information about which options are likely to be in the top four. From Figure 3, D, F, C, and A are more likely than not to be in the top four at λ = £20,000, and it is also noteworthy that these form a fairly tight cluster on the cost‐effectiveness plane (Figure 1). Other therapies are more likely than not to be in the “bottom three” for net benefit at λ = £20,000, either because of lack of effectiveness (B and E) or high costs (G). A promising area of qualitative research might be to study decision makers' objectives and attitude to risk when faced with an MTA and whether rank probabilities are informative or useful alongside conventional ways of communicating results.

NMA usually synthesizes clinical outcomes but shares many of the mathematical properties of an economic evaluation (particularly, the assumption of transitivity and an interpretation that fits more naturally into a Bayesian framework). Given the increasing acceptability of NMA, the use of similar presentational devices such as rank probabilities in the evidence synthesis and economic analyses makes HTA as a whole more coherent.

To the author's knowledge, the use of rank metrics in economic evaluation has not been proposed before. It is hoped that this paper will stimulate discussion, initiate further methodological research, and increase awareness among decision makers of their interpretation and usefulness.

## CONFLICTS OF INTEREST

No funding has been received and no conflict of interest is reported.

## Supporting information


**Table S1**. Model inputs and mean outputs for the varicose veins case study.
