# Supplementary material for: Beyond the cost‐effectiveness acceptability curve: The appropriateness of rank probabilities for presenting the results of economic evaluation in multiple technology appraisal
Source: Health Econ. 2019 May 2;28(6):801–7. doi: 10.1002/hec.3884 (PMC6790661; doi:10.1002/hec.3884)
Supplement: Supplementary file 1 — Table S1. Model inputs and mean outputs for the varicose veins case study. [file HEC-28-801-s001.docx]

Supplementary Material

Case study

A case study is used to illustrate and motivate the discussion. The patients are people with severe symptomatic varicose veins(National Clinical Guideline Centre 2012). The costs and QALYs were generated by a simple stochastic model, available on a CC BY 4.0 license <http://dx.doi.org/10.17632/pmrt2gnzcr.1>. The input data to the model are stylized but are intended to be fairly realistic(Epstein et al. 2018). The model is a decision tree with a 5 year time horizon. To simplify, no discounting is applied. There are 7 therapies, labeled A – G. A is the standard interventional treatment, taken as the reference or “comparator”. B is conservative care, that is, no interventional treatment, with no initial cost. The others are competing interventional therapies with procedure cost (*c_proc_j_*) given in Supplementary Table S1. The (binary) measure of effectiveness is re-intervention. A re-intervention would only be considered if the index procedure has failed. In this case the patient will have continued to experience severe symptoms from the time of the index procedure up to the date of re-intervention. The risk ratios and confidence intervals are given in Supplementary Table S1 (they could have been estimated by NMA, for example). If no re-intervention is needed, the procedure is deemed successful and the patient has full quality of life. Re-intervention is undertaken using procedure “E” (cost £249). While waiting for the re-intervention (six month waiting list), the patient will have symptoms with EQ-5D health-related quality of life (HRQOL) of *u*= 0.64 (SE 0.04)(Clegg and Guest 2007). The log-incidence of re-intervention under procedure A is ln*r_a_*=-3.38 (SE 0.245). The 5 year probability of re-intervention is calculated as *R_j_* = 1- exp(-5×exp(ln*r_a_*)×*rr_j_*), where *rr_j_* is the risk ratio for *j* versus treatment A. For the PSA, risk ratios have log-normal distributions, procedure costs have gamma distributions and the HRQOL decrement (1-*u*) has a gamma distribution(Briggs, Claxton, and Sculpher 2006). Given these data, total costs over 5 years will be *C_j_*=*c_proc_j_*+*R_j_*×249, and QALY *Q_j_* = 5 – (1-*u*)×*R_j_*×0.5

Supplementary Table S1. Model inputs and mean outputs for the varicose veins case study

|  | Cost of procedure, mean (SE) | Risk ratio of re-intervention | 5 year probability of re-intervention, derived from rate in therapy A and risk ratio | Mean total cost over 5 years | Mean total QALY over 5 years |
| --- | --- | --- | --- | --- | --- |
| A | 894 (100) | 1 (reference treatment) | 0.137 | 935 | 4.971 |
| B | 0 | 7.52, 1.22-46.37 | 0.871 | 169 | 4.878 |
| C | 743 (100) | 1.13, 0.72- 1.77 | 0.166 | 791 | 4.967 |
| D | 767 (100) | 0.48, 0.18-1.29 | 0.338 | 792 | 4.984 |
| E | 249 (50) | 4.93, 2.59-9.37 | 0.494 | 388 | 4.899 |
| F | 862 (100) | 0.46,0.01-29.92 | 0.566 | 913 | 4.964 |
| G | 1317 (100) | 1.00, 0.01-29.92 | 0.004 | 1392 | 4.945 |

Note. A is the reference treatment, and B is “conservative care”. Expected cost and QALY calculated as the mean over 1000 Monte – Carlo simulations

References

Briggs, A, Karl Claxton, and Mark Sculpher. 2006. *Decision Modelling for Health Economic Evaluation*. Oxford: OUP.

Clegg, J P, and J F Guest. 2007. “Modelling the Cost-Utility of Bio-Electric Stimulation Therapy Compared to Standard Care in the Treatment of Elderly Patients with Chronic Non-Healing Wounds in the UK.” *Curr Med Res Opin* 23 (4): 871–83. https://doi.org/10.1185/030079906x167705.

Epstein, D., S. Onida, R. Bootun, M. Ortega-Ortega, and A.H. Davies. 2018. “Cost-Effectiveness of Current and Emerging Treatments of Varicose Veins.” *Value in Health*. https://doi.org/10.1016/j.jval.2018.01.012.

National Clinical Guideline Centre. 2012. “Varicose Veins in the Legs.” *National Guideline*. https://doi.org/10.1017/CBO9781107415324.004.
